# Comparison of the Accuracy of 2D and 3D Templating for Revision Total Hip Replacement

**DOI:** 10.3390/jpm13030510

**Published:** 2023-03-12

**Authors:** Philipp Winter, Ekkehard Fritsch, Jochem König, Milan Wolf, Stefan Landgraeber, Patrick Orth

**Affiliations:** 1 Department of Orthopaedic Surgery, University of Saarland, Kirrberger Straße, 66421 Homburg, Germany; 2 Institute of Medical Biostatistics, Epidemiology and Informatics, University Medical Centre of the Johannes Gutenberg University, 55131 Mainz, Germany

**Keywords:** revision arthroplasty, hip (joint), reinforcement cage, Burch Schneider, 2D, 3D, computed tomography

## Abstract

Introduction: Revision hip arthroplasty is a challenging surgical procedure, especially in cases of advanced acetabular bone loss. Accurate preoperative planning can prevent complications such as periprosthetic fractures or aseptic loosening. To date, the accuracy of three-dimensional (3D) versus two-dimensional (2D) templating has been evaluated only in primary hip and knee arthroplasty. Methods: We retrospectively investigated the accuracy of 3D personalized planning of reinforcement cages (Burch Schneider) in 27 patients who underwent revision hip arthroplasty. Personalized 3D modeling and positioning of the reinforcement cages were performed using computed tomography (CT) of the pelvis of each patient and 3D templates of the implant. To evaluate accuracy, the sizes of the reinforcement cages planned in 2D and 3D were compared with the sizes of the finally implanted cages. Factors that may potentially influence planning accuracy such as gender and body mass index (BMI) were analyzed. Results: There was a significant difference (*p* = 0.003) in the accuracy of correct size prediction between personalized 3D templating and 2D templating. Personalized 3D templating predicted the exact size of the reinforcement cage in 96.3% of the patients, while the exact size was predicted in only 55.6% by 2D templating. Regarding gender and BMI, no statistically significant differences in planning accuracy either for 2D or 3D templating were observed. Conclusion: Personalized 3D planning of revision hip arthroplasty using Burch Schneider reinforcement cages leads to greater accuracy in the prediction of the required size of implants than conventional 2D templating.

## 1. Introduction

The number of primary hip arthroplasties is steadily increasing, and it is inevitable that revision of failed hip replacements will become more frequent in the future [1]. The most common reasons for revision are aseptic loosening, instability and periprosthetic infection [2,3]. It is always a challenging procedure, especially in cases of severe bone loss, and preoperative planning is therefore of utmost importance, and indispensable if accurate results are to be achieved [4]. It is an established fact that preoperative planning in primary hip arthroplasty can reduce the rate of complications due to over- or underestimation of implant size [5,6] as well as lead to a reduction in surgery time and an improvement in postoperative stability and range of motion [7]. At the same time, it can reduce costs because it renders large inventories of implants unnecessary [8]. Templating also has an important and growing legal significance in today’s world. By templating, the surgeon can prove that they have already thought through the procedure preoperatively. A potential imputation of negligence can thus be averted.

With the introduction of picture archiving and communication systems (PACS), the image size of radiographs is no longer standardized and therefore needs to be calibrated [9]. According to Sinclair et al., incorrect positioning of the calibrating sphere results in a mean percent error in template creation of 6.8% (range 0–26%) [10]. Other studies have reported increased inaccuracy of the magnification factor in obese patients [11]. To improve the accuracy of preoperative planning, three-dimensional (3D) templating has been compared with two-dimensional (2D) templating in primary hip arthroplasty in recent years [12,13]. A systematic review by Bishi et al. demonstrated the greater accuracy and reliability of 3D templating in predicting the required implant size in preoperative planning of primary arthroplasty compared with 2D templating [13]. Here, different 3D templating methods were compared, based on either a CT data set or biplanar radiographs. So far, there have been no studies focusing on the accuracy of personalized 3D templating in revision arthroplasty, a procedure for which preoperative surgical planning is of especially crucial importance. Reliable prediction of the exact implant size before surgery can increase intraoperative safety and reduce inventory costs. Therefore, this study aimed to evaluate the accuracy of personalized computed tomography by 3D templating in comparison to conventional 2D templating in revision hip arthroplasty using reinforcement cages.

## 2. Materials and Methods

In this retrospective study, we included all patients with sustained massive bone defects (Paprosky type 2A–3B) who underwent revision hip arthroplasty with implantation of a reinforcement cage by senior surgeons at our institution between April 2019 and November 2022. Patients who did not have a reinforcement cage implanted or did not have preoperative computed tomography were excluded.

The study respected the ethical standards for biomedical research in accordance with the Declaration of Helsinki [14], met the ethical review requirements for our institution and was approved by the local Ethics Committee (reference number 254/22).

### 2.1. Preoperative Imaging

**Figure 1:**
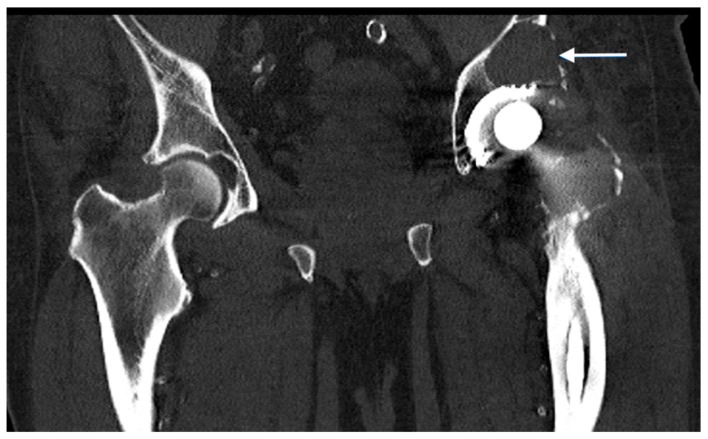
Computed tomography of a patient with an aseptic loosening of a hip endoprosthesis and an acetabular abrasion granuloma (indicated by the white arrow).

Standard conventional radiography was performed for all patients in an anteroposterior (A/P) view of the pelvis and a lateral view of the hip in the Lauenstein position. In addition, preoperative computed tomography (CT) of the pelvis was routinely performed to assess the bone defect situation and to plan the operative procedure. A helical CT scanner with a slice thickness of 0.75 mm was used. The CT data were transferred in our PACS system Sectra, Sweden (Sectra AB, Linköping, Sweden) (Figure 1).

### 2.2. Digital Templating

**Figure 2:**
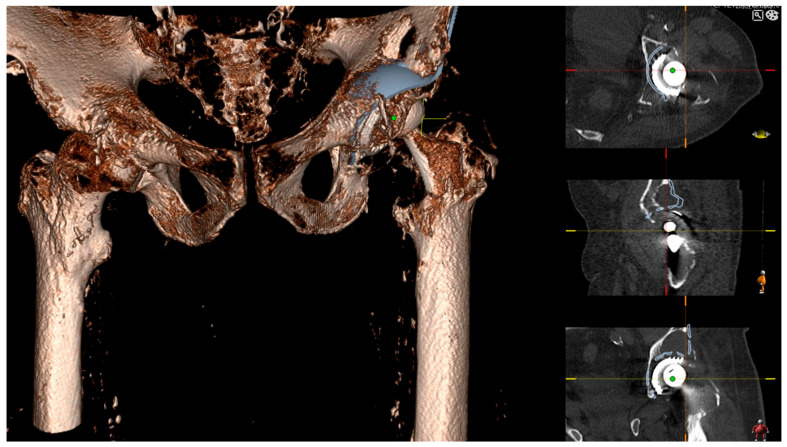
Personalized 3D templating using 3D Joint Sectra (Sectra AB, Linköping, Sweden) planning software (version 24.2.6). Planning aimed to bridge the granuloma in the area of the acetabular dome using the proximal flange of the Burch Schneider reinforcement cage.

**Figure 3:**
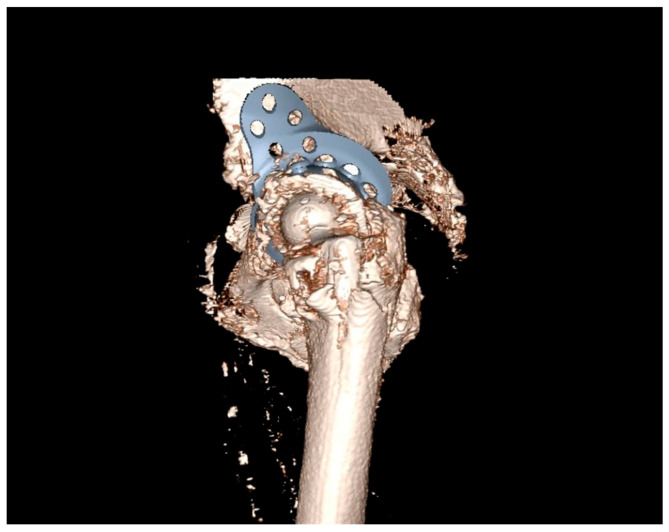
Lateral view to check correct alignment and sufficient bridging of the bone defects.

**Figure 4:**
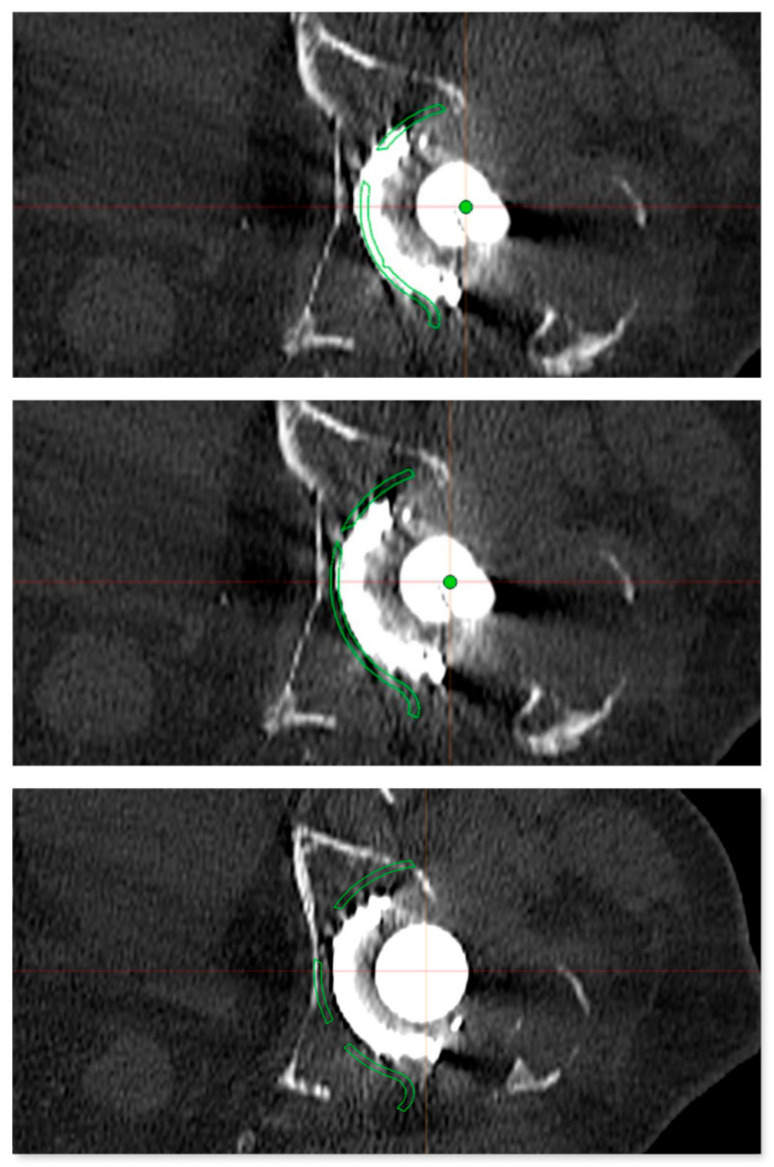
Preoperative planning of the reinforcement cage (Burch Schneider) in the transversal plane of the CT scan. The results of the personalized planning are shown in the center image, indicative of a Burch Schneider reinforcement cage 56 mm in diameter. The upper and lower images show reinforcement cages one size smaller (50 mm; top) and one size larger (62 mm, bottom). In this patient, a 56 mm size reinforcement cage was implanted.

Three-dimensional modeling and templating of the reinforcement cage was based on computed tomography imaging analyses of the pelvis (Figure 2, Figure 3 and Figure 4). Conventional 2D templating was always performed preoperatively according to the X-ray images in two planes. To determine the magnification factor, a radiopaque metal ball with a standardized diameter of 25.0 mm was used as a reference and placed between the legs of the patient at the level of the hip joint rotation center. Preoperative 2D planning with the Sectra (Sectra AB, Linköping, Sweden) 2D planning system was performed by senior surgeons using the 2D templates of the reinforcement cage. With the help of the IT solution for medical imaging, Sectra (Sectra AB, Linköping, Sweden), 3D templating was performed by applying the 3D Sectra Joint Replacement Tool with the corresponding 3D templates of the reinforcement cage (Burch Schneider, Zimmer Biomet, Warsaw, IN, USA). Three-dimensional templating was performed independently of the previous 2D templating and was compared with the implanted cage size by an independent examiner. All 2D preoperative templating was performed by three senior surgeons. Personalized 3D templating was performed by a resident with the support of the senior surgeons.

### 2.3. Surgical Procedure

**Figure 5:**
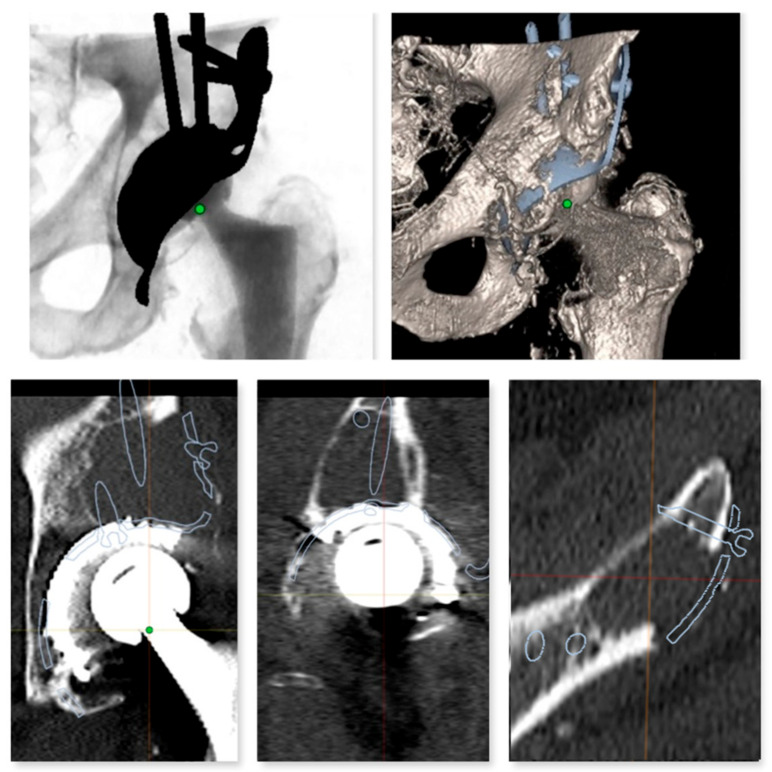
Planning of the screw fixation of the Burch Schneider reinforcement cage. The optimal screw positioning was preoperatively planned by 3D CT imaging.

**Figure 6:**
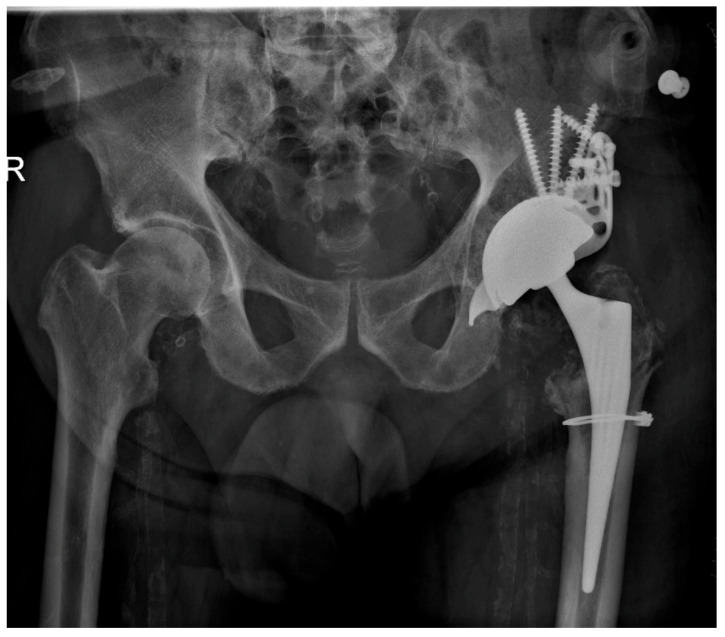
Postoperative result after implantation of a Burch Schneider reinforcement cage (diameter 56 mm). The periprosthetic fracture of the femur (Vancouver A2) was fixed with a cerclage wire.

All patients were operated on through a lateral surgical approach in the lateral position. The reinforcement ring used in all cases was a Burch Schneider cage (Zimmer Biomet, Warsaw, IN, USA). Preexisting acetabular defects were classified according to the Paprosky system [15] and are shown in Table 1. The main objective was to anchor the Burch Schneider cage as stably as possible in the correct position within the host bone. Restoration of natural biomechanical conditions such as leg length, center of rotation and lateralization were additional major aims of the surgical procedure. The Burch Schneider cage was secured at the acetabular roof by at least three pile screws and three horizontal screws (Figure 5 and Figure 6). For medial and caudal fixation, the distal nose of the cage was tapped into the os ischii using a specific opening chisel. A cemented dual-mobility cup (Avantage, Zimmer Biomet, Warsaw, IN, USA) was implanted into the reinforcement ring in all cases. The cemented cup was positioned within the Lewinnek safety zone of 40 ± 10° inclination and 15 ± 10° anteversion.

### 2.4. Statistical Analysis

Statistical analyses were conducted using the SPSS software package (version 29; IBM SPSS Statistics, Chicago, IL, USA). The two-sided significance level was *p* < 0.05. Systematic differences between 2D and 3D templating and the intraoperatively used implant size on the other hand side was assessed by means of contingency tables and the exact Wilcoxon signed-rank tests. The proportion of correct size predictions by planning is referred to as accuracy and compared between 2D and 3D templating using McNemar’s test. The influence of the patient’s sex on the accuracy of templating was tested using Fisher’s test for 2 × 2 contingency tables. The influence of BMI on planning accuracy was tested using the Mann-Whitney U test for comparing patient groups with correctly to incorrectly predicted sizes with respect to BMI.

## 3. Results

Strictly applying the inclusion and exclusion criteria, 27 cases were finally included in this study. Fifty-six percent (*n* = 15) were female and forty-four percent (*n* = 12) were male patients. The overall mean age was 72.3 (±11.9) years. The mean age of female patients was 76.4 (±6.7) years, and that of male patients was 67.3 (±15.2) years. The demographic data of the patients are shown in Table 1. According to the World Health Organisation (WHO) criteria [16], one patient was underweight, six patients were in the normal range, eleven patients were overweight, and nine patients were obese. The mean time between the index operation and the revision arthroplasty was 12.7 years (152.3 ± 141.9 months standard deviation). Acetabular bone defects were classified according to the Paprosky classification. A total of 22 patients were graded as Paprosky type II defect (six with IIA, 11 with IIB and five with IIC) and five patients as Paprosky type III defect (two with IIIA and three with IIIB), as shown in Table 1.

The exact size of the reinforcement cage as determined intraoperatively was correctly predicted in 26/27 patients with personalized 3D templating, and in only 15/27 patients with conventional 2D templating according to the initial implant sizes as determined intraoperatively (McNemar test *p* = 0.0034). 2D templating underestimated the intraoperatively determined size more frequently than overestimating it (eight vs. four times, Table 2), but this was not statistically significant (exact Wilcoxon signed rank test *p* = 0.12). Three-dimensional templating underestimated the intraoperatively determined size in one instance and never overestimated it (Table 3, exact Wilcoxon signed rank test *p* = 1). Surprisingly, the only patient with size incorrectly predicted by 3D planning had the size correctly predicted by 2D planning (Appendix A).

### 3.1. Gender and Planning Accuracy

Compared with the intraoperatively implanted reinforcement cages, conventional 2D templating of the implants was correct in 46.7% (*n* = 7/15) of female patients and in 41.7% (*n* = 5/12) of male patients (Fisher’s exact test *p* = 1). Personalized 3D templating was able to predict the correct implant size in all male patients (*n* = 12/12) and in 93.3% (*n* = 14/15) of females (Fisher’s test *p* = 1).

### 3.2. BMI and Planning Accuracy

Patients were classified into four BMI groups according to WHO criteria: underweight, normal weight, overweight, and obese (Table 1). The accurate size of the reinforcement cage was predicted by personalized 3D templating in 1/1 for underweight, 4/5 normal weight, 11/11 overweight and in 10/10 obese patients. In 2D templating, the accurate size was predicted in 1/1 underweight, 4/5 normal weight, 2/11 overweight, and in 8/10 obese patients. The Mann–Whitney U test revealed no statistically significant difference in BMI between correctly and incorrectly predicted size for 2D templating and 3D templating (*p* = 0.35 and *p* = 0.44, respectively).

## 4. Discussion

One of the most important aspects of preoperative planning for an acetabular revision prosthesis in hip arthroplasty is meticulous templating based on X-rays and other imaging analyses such as CT scans. The major goal of preoperative planning is to accurately predict the size and optimal position of the implants prior to the surgical procedure. In primary arthroplasty, templating has been proven to reliably predict implant dimensions to within one size and reduce the rate of intraoperative and postoperative complications [5,6,7,13]. A systematic review demonstrated that 3D templating is more accurate than 2D templating in the planning of primary total hip arthroplasty [13]. Until now, however, there have been no studies on the accuracy of 3D templating in revision hip arthroplasty. Therefore, we retrospectively compared the accuracy of 2D and 3D templating in 27 cases of hip revision arthroplasty using Burch Schneider reinforcement cages. Our results revealed an accuracy of 56% for conventional 2D templating and of 96% for personalized 3D templating.

There is little literature available to date regarding the accuracy of preoperative planning for revision arthroplasty. Most studies on preoperative planning in revision arthroplasty focus on 3D printing or patient-specific implants [17,18,19]. However, these two procedures are associated with significantly higher costs [20,21,22]. In primary hip arthroplasty, a cost analysis of 3D templating identified an additional cost of 53–116 € per patient. In this study, the direct costs of preoperative computed tomography mainly include the direct fixed cost for CT scanning and the cost for personnel involvement [23]. As computed tomography was routinely performed as part of our study, the cost for this procedure can be ignored. Nevertheless, the cost for 3D templating must be taken into account, especially the personnel cost. Personnel involvements of 16 min for the surgeon were determined by a cost analysis in 3D templating for primary hip arthroplasty [23]. Especially in revision arthroplasty, a higher time expenditure for 3D templating can be assumed compared to primary arthroplasty. On the other hand, time can be saved in 2D planning, which may become obsolete in the future. Future studies should investigate the time required for 3D templating in revision arthroplasty, and a cost analysis should be performed.

Maryada et al. were able to simulate and plan the correct implant size and positioning of the acetabular cup through printed anatomical 3D models. Accurate placement of the acetabular cup in complex primary and revision total hip replacement was achieved in 93% of cases (*n* = 27) [17]. These findings match our results for 3D templating of reinforcement cages. The 3D printed model can also be used to simulate the required position and length of acetabular screws [17]. Personalized 3D digital templating also offers this advantage. Here, just as with 3D printing, a 3D virtual image is created and enables a valid simulation of the implantation of the reinforcement cage. However, any tactile sensation that might be achieved by 3D printing cannot be simulated by digital solutions. In the future, optical see-through devices could be used to project a hologram of the personalized 3D model onto the surgical site and simulate the correct size and positioning. Initial experimental studies have already been conducted in this area, but are currently limited to primary arthroplasty [24,25,26].

With regard to BMI, no statistically significant effect was found for 2D and 3D templating accuracy for implantation of reinforcement cages in obese patients, which is in line with the results of Heep et al. and Holzer et al. [27,28]. Holzer et al. reported no statistically significant impact of BMI on the planning accuracy for the acetabular cup. However, statistically significant differences between normal and overweight patients were observed in the planning accuracy for the femoral components [27]. Heep et al. could not demonstrate a correlation between body shape parameters such as BMI and the magnification of a radiopaque reference object [28]. In our study, there was no statistically significant difference in the accuracy of either 2D or 3D templating between normal and overweight patients. In overweight and obese patients, a negative influence on the planning accuracy in 2D templating would theoretically be possible due to the magnification error. Such a magnification error is not expected in patient-specific 3D templating due to the routinely performed computed tomography. However, our study did not detect any effect of BMI on the planning accuracy for either conventional 2D or patient-specific 3D templating.

No gender-specific differences were found in the planning accuracy of 2D and 3D templating. Consequently, the reliability of preoperative personalized 3D templating can be assumed for both women and men. This, again, is consistent with the findings of Heep et al. and Holzer et al., who also found no statistically significant gender-specific differences in planning accuracy, and no statistically significant deviation in calibration of X-rays between men and women [27,28].

In conclusion, we increased the accuracy of size prediction for reinforcement rings in revision hip arthroplasty by using a personalized digital planning tool. The greater accuracy of personalized 3D templating identified in our study can probably be attributed to the use of actual-size images and independence from patient positioning and rotation [29,30]. The retrospective design of the current study has the advantage that the personalized 3D planned implant size and the actual implant size were determined independently of each other and by an independent examiner. The influence of the 2D templating on the 3D templating could be prevented by the retrospective design of the study. In a prospective study, attention will be paid to this circumstance to prevent a possible influence of the respective planning. Limitations of this study include the lack of calculation of intra- and interobserver reliability. Secondly, we only investigated one reinforcement cage design (Burch Schneider). This may have allowed easier templating compared with other reinforcement cages. Finally, the increased accuracy of 3D templating has not yet been sufficiently analyzed to be able to identify any benefit on the clinical outcome of the surgical procedure, and the additional cost needs to be determined.

## 5. Conclusions

This study shows that 3D templating based on computed tomography is a reliable tool for personalized planning in revision hip arthroplasty using Burch Schneider reinforcement cages, and leads to greater accuracy in the prediction of the required implant size than conventional 2D templating. Precise planning of screw positioning is also conceivable with the appropriate 3D templates and provides valuable additional preoperative information regarding the most stable fixation of the reinforcement cage. Further prospective studies will shed more light on the importance of customized prostheses when standard designs are compared during 3D templating for the same patient.

## Figures and Tables

**Table 1:** Patients’ demographic data and Paprosky classification of the acetabular bone loss.

Age (mean ± SD)	72.3 ± 11.9
Female	15
Male	12
BMI (WHO classification in kg/m^2^)	
underweight (<18.5)	1
normal weight (18.5–24.9)	5
overweight (≥25.0)	11
obese (≥30.0)	10
Paprosky classification of acetabular bone loss	
Paprosky type 2A	6
Paprosky type 2B	11
Paprosky type 2C	5
Paprosky type 3A	2
Paprosky type 3B	3

SD—standard deviation.

**Table 2:** Accuracy of 2D templating comparing different implant sizes of the reinforcement cage.

			2D Templating				Total
			44 mm	50 mm	56 mm	62 mm	
Intraoperative size	50 mm		1	4	0	0	5
	56 mm		0	4	9	3	16
	62 mm		0	1	3	2	6
Total			1	9	12	5	27

**Table 3:** Accuracy of 3D templating comparing different implant sizes of the reinforcement cage.

			3D Templating			Total
			50 mm	56 mm	62 mm	
Intraoperative size	50 mm		5	0	0	5
	56 mm		1	15	0	16
	62 mm		0	0	6	6
Total			6	15	6	27

## Data Availability

The data used to support the findings of the present study are available from the corresponding author upon request.

## Appendix A

Showing the planned size of the reinforcement cage by 2D templating and by 3D templating of each patient, and the final size of the reinforcement cage.

**Table:**

		2D Templating	Final Implant	3D Templating
**Patient number**	1	**62 mm**	56 mm	56 mm
	2	56 mm	56 mm	56 mm
	3	56 mm	56 mm	56 mm
	4	56 mm	56 mm	56 mm
	5	50 mm	50 mm	50 mm
	6	56 mm	56 mm	56 mm
	7	56 mm	56 mm	56 mm
	8	**44 mm**	50 mm	50 mm
	9	**50 mm**	56 mm	56 mm
	10	**50 mm**	56 mm	56 mm
	11	50 mm	50 mm	50 mm
	12	56 mm	56 mm	56 mm
	13	62 mm	62 mm	62 mm
	14	**62 mm**	56 mm	56 mm
	15	**50 mm**	56 mm	56 mm
	16	56 mm	56 mm	56 mm
	17	**50 mm**	62 mm	62 mm
	18	50 mm	50 mm	50 mm
	19	**56 mm**	62 mm	62 mm
	20	**50 mm**	56 mm	56 mm
	21	56 mm	56 mm	**50 mm**
	22	56 mm	56 mm	56 mm
	23	**56 mm**	62 mm	62 mm
	24	62 mm	62 mm	62 mm
	25	**56 mm**	62 mm	62 mm
	26	**62 mm**	56 mm	56 mm
	27	50 mm	50 mm	50 mm
		54.6 mm	56.2 mm	56.0 mm
